# The Hygiene of Transmissible Diseases: Their Causation, Modes of Dissemination and Methods of Prevention

**Published:** 1899-10

**Authors:** 


### Book Reviews.

THE HYGIENE OF TRANSMISSIBLE DISEASES: Their Causation, Modes of Dissemination and Methods of Prevention. By A. C. ABBOTT, M. D., Professor of Hygiene and Director of the Laboratory of Hygiene, University of Pennsylvania. Octavo volume of 311 pages. Illustrated. Philadelphia: W. B. Saunders, Publisher.

In an address delivered before the Medical and Chirurgical Faculty of Maryland, at the celebration of the centennial anniversary of its foundation, April 26, 1899, Dr. W. W. Keen, of Philadelphia, said that "public hygiene or sanitation has been a very large element in arresting the ravages of disease which in former times swept over entire nations, and even continents; and it is a source of pride to us that among the foremost sanitarians in every community are the doctors. It is a very striking fact that diseases which once assumed the form of veritable pestilences are now, at least in civilised countries, almost unheard of; and others, though they have not yet disappeared, have had their fangs drawn, so that the public suffers far less than it formerly did. If the voice of the profession were heeded, even the diseases which have been largely abated would almost, if not entirely disappear."

The importance of public hygiene at the present time and of popular teaching of this subject in our medical colleges is becoming apparent to every careful observer who has watched the ravages of transmissible diseases for the past ten years. The bubonic plague in India, the cholera epidemic in Hamburg and the lesser epidemics of yellow fever, small-pox and typhoid fever in our own country have put physicians and hygienists on their guard and the opening sentences of Professor Keen's address are not inappropriately quoted in calling attention to Dr. Abbott's timely work on this subject. Preventive medicine is doomed to be the great study of the coming century, not to the physician alone, but every intelligent man, for his own personal welfare as well as that of the community, should be familiar with sanitary laws, especially those concerning the causation and spread of disease and the means of prevention. He should be familiar with the modes of infection the methods of disinfection, the means for the isolation of the sick, and the general rules for the management of contagious disease.

This important and necessary information the present volume seeks to supply. It is not the purpose of the book to present a complete survey of the entire field of hygiene; but it deals with just that practical portion of the subject which is of vital interest to every intelligent man, be he physician or layman, who has at heart his own best interests as well as those of the community of which he forms a part.

The author in the introductory chapter discusses the predisposing and exciting causes of disease, such as age, sex, race, occupation, heredity, season, density of population, and such exciting causes as malnutrition, malformation, other defects of metabolism and physiological function, excessive heat, cold, moisture and bacteria. The postulates of Koch's demonstrating a causal relation between a given bacterial species and disease is given an important place in this chapter.

This chapter is supplied with various interesting charts, showing by months the average relative proportions of deaths from diphtheria and scarlatina in Philadelphia; the seasonal variation in the occurrence of typhoid fever and of malarial fever; the monthly fluctuations of diarrheal troubles, also the death-rates from diseases of the respiratory system in New York City.

Section II. is devoted to transmissible diseases, giving a summary of points that are of importance to the hygienist, such as the causation, modes of dissemination, portals of infection, prophylaxis against, and the geographical distribution of such diseases. Under this head are discussed typhoid fever, Asiatic cholera, amebic dysentery, tuberculosis, diphtheria, influenza, bubonic plague, leprosy, lock-jaw, anthrax, glanders, actinomycosis, small-pox, measles, varicella, scarlet fever, whooping cough, mumps, malarial fever, dengue, typhus and relapsing fevers, rabies and diseases due to animal parasites. These chapters are of vital interest to physicians and are interspersed with valuable data regarding various outbreaks, especially the cholera outbreak in Hamburg and Altona and the recent epidemic of bubonic plague in Hong Kong. On the subject of medical examination of prostitutes, the author shows that in the various countries where the contagious diseases act is in operation the percentage of syphilis among women has materially decreased. The author speaks very forcibly on this point, advocating inspection as follows: "To many it may appear improper to advocate a method by which the community acknowledges and openly licenses a practice that is plainly a breach of public morals. But the evil exists; it is likely to exist and nothing is gained, on the contrary much is lost, by calmly closing the eyes to it."

Section III is devoted to prophylaxis in general against infectious diseases, including vital, chemical and physical processes, the management of contagious diseases and quarantine. In this chapter the author discusses immunity, natural and acquired, the practice of vaccination and protective inoculation and the evolution of our knowledge of the antitoxic condition. Regarding antitoxin in general the author says (page 248): "In the present state of our knowledge it is impossible to say to what extent acquired immunity in human beings is due to the presence of antitoxic substances in the circulating fluids, or to indicate in how far the observations that have been made upon tetanus and diphtheria are applicable to other infections; certainly in so far as the truly toxic infections are concerned, one is constrained to feel sanguine as to the ultimate outcome of the further application of the principles on which the antitoxic method of treatment is based."

Under the chemical prophylactic measures the author gives the essenitals of a useful and a trustworthy disinfectant: (1) It shall be a germicide, *i. e.*, it shall possess the property of destroying bacteria and their spores; (2) It should be so constituted that its germicidal properties are not destroyed by the extraneous matters in which the infective microörganisms that are to be killed are to be located; (3) With ordinary care it should not be dangerous—directly poisonous—to those who are to use it; (4) It should if possible be without disagreeable odor; it should be cheap in price, easy to manipulate and for particular purposes should not cause permanent stains or be destructive to the skin, to fabrics, or to other articles on which it is employed.

The author describes the various agents used as disinfectants, giving preference to formalin, to which 10 per cent. of glycerin has been added and through the employment of a generator, similar to the Novy-Waite apparatus, the gas is disengaged by heat and conveyed through a tube into the room to be disinfected. The disagreeable and irritating odors of the formaldehyde may be removed by sprinkling the room with ammonia water.

The disinfection of water-closets, urinals and sinks, stables and cellars, wells and cisterns are then taken up separately and specifically considered.

Welch's and Fürbringer's methods of disinfecting the hands as developed at the Johns Hopkins Hospital are given in full. The author then discusses the physical processes of disinfection, as through heat, desiccation, sunlight, electricity and mechanical agitation.

The closing chapters are devoted to isolation, the care of the sick-room, clothing, excreta, utensils, attendants, the care of the body after death and a careful consideration of quarantine.

On the whole, apart from its scientific value, the book is highly interesting and entertaining. The meaning of the charts is shown at a glance and the photomicrographs well executed. The publishers have done their work admirably. W. C. K.

